# Changes in Body Compositions and Basal Metabolic Rates during Treatment of Graves' Disease

**DOI:** 10.1155/2018/9863050

**Published:** 2018-05-03

**Authors:** Min Joo Kim, Sun Wook Cho, Sumin Choi, Dal Lae Ju, Do Joon Park, Young Joo Park

**Affiliations:** ^1^Department of Internal Medicine, Seoul National University Hospital, Seoul, Republic of Korea; ^2^Department of Food Service and Nutrition Care, Seoul National University Hospital, Seoul, Republic of Korea; ^3^Department of Internal Medicine, Seoul National University College of Medicine, Seoul, Republic of Korea

## Abstract

**Objectives:**

Because thyroid hormone is an important determinant of body weight and basal metabolic rate, we investigated the changes in the basal metabolic rate and body composition sequentially after treatment for Graves' disease.

**Methods:**

A prospective cohort study was performed with six women newly diagnosed with Graves' disease. During a 52-week treatment of methimazole, body composition, resting respiratory expenditure (REE), and handgrip strength were measured consecutively.

**Results:**

After methimazole treatment, body weight was initially increased (0–8 weeks), subsequently plateaued (8–24 weeks), and gradually decreased in the later period (24–52 weeks) despite the decreased food intake. The measured REE was 40% higher than the predicted REE at baseline, and it gradually decreased after treatment. REE positively correlated with thyroid hormone levels, peripheral deiodinase activity, and thyroid's secretory capacity. Body compositional analyses showed that the fat mass increased during an earlier period (4–12 weeks), while the lean mass increased significantly during the later period (26–52 weeks). Consistent with the lean mass changes, muscle strength also significantly increased during the later period.

**Conclusions:**

Treatment of Graves' disease increased body weight and fat mass transiently with decreased REE. However, long-term compositional changes moved in a beneficial direction increasing lean mass and reinforcing muscle strength, following decreasing fat percentages.

## 1. Introduction

The thyroid hormone is an important determinant of the body composition by regulating the basal metabolic rate and thermogenesis [1]. Increased basal metabolic rate or resting energy expenditure has been observed in patients with hyperthyroidism [2, 3]. Several studies have shown that hyperthyroidism-related weight loss reflects not only the depletion of body fat but also the loss of muscle mass in association with an accelerated whole body catabolism following increased thermogenesis and oxygen consumption [4]. Indeed, weight loss, despite the increased appetite and food intake, is one of the clinical manifestations of untreated hyperthyroidism. Increased food intake observed in patients with hyperthyroidism is not only a result of energy consumption but also a direct consequence of it affecting the appetite-regulating hypothalamic nuclei [2, 5].

Meanwhile, treatment of hyperthyroidism consequently increases body weight and changes the body composition [4, 6, 7]. Whether it is a balanced weight gain composed of fat and lean mass is undetermined. Conflictingly, lean mass [6], fat mass [7], or both of them [3, 4] have been reported as the major component of the treatment-related weight gain in hyperthyroidism.

The earlier experience of weight loss following rapid weight gain during antithyroid treatment can give patients a reasonable concern whether these treatment-related changes are healthy or not. The aim of this study was to investigate the change in body composition and metabolic rates consecutively during the treatment of hyperthyroidism.

## 2. Materials and Methods

### 2.1. Subjects

Newly diagnosed Graves' disease patients were recruited between June 2012 and February 2013. Graves' disease was diagnosed based on clinical examination with laboratory data (suppressed thyrotropin [TSH] with high free thyroxine [fT4] levels), increased diffuse thyroid uptake of 99mTc in the radionuclide scintigraphy, and/or the present of TSH receptor antibody. Eight female patients were screened and 6 were enrolled. Two patients were excluded because of their intolerance to the resting metabolic rate (RMR) test. All 6 patients were treated with antithyroid drugs to achieve euthyroidism. Informed consent was obtained from all patients. The study protocol was approved by the Institutional Review Board of Seoul National University Hospital (1303-046-473).

### 2.2. Assessment of Dietary Intake and Quality of Life

To assess the dietary intake, a three-day dietary record was used. Before each clinic visit, patients were asked to record the type and amount of each food consumed for 3 days (two weekdays and one weekend day). Total energy and nutrient intake were calculated using the nutritional analysis program (CAN-Pro) version 5.0 (Korean Nutrition Society, Seoul, Korea). In addition, the estimated energy requirement was calculated according to the Korean Dietary Reference Intakes (2015) by the Korean Nutrition Society using the following formula >[8]: Estimated energy requirement = 354 − 6.91 × age(years) + 1.12 × [9.36 × body weight(kg) + 726 × height(m)]. To evaluate the quality of life, SF-36 was surveyed before and after 52 weeks of treatment. The score was converted into 100 points.

### 2.3. Measurement of Body Composition, Resting Metabolic Rates, and Muscle Strength

Height, weight, waist circumference, hip circumference, body composition, blood pressure, and heart rate were measured at each clinic visit. Body composition including lean mass, fat mass, and fat content was measured by bioelectrical impedance with the body composition analyzer (InBody 720, Biospace, Seoul, Korea). Dual-energy X-ray absorptiometry (DXA; GE Lunar Prodigy, Madison, WI, USA) was used to analyze the body composition and bone mineral density at 0 and 52 weeks.

After an 8~14 h overnight fast, the RMR test was performed using the breath-by-breath pulmonary gas exchange (Quark b2, COSMED, Rome, Italy) at baseline and at 4, 8, 12, 26, and 52 weeks after treatments. Respired gases (oxygen and carbon dioxide) were analyzed, and resting energy expenditure (REE) was calculated indirectly. The predicted REE was calculated as follows: (665.096 + 9.563 × weight + 1.85 × height) − 4.565 × age [9].

Muscle strength was evaluated with an electronic handgrip dynamometer (Lavisen, Namyangju, Korea) because it is feasible, convenient, and inexpensive. Patients were asked to grasp the dynamometer at their maximal power in a sitting position with an adducted and neutrally rotated shoulder, a 90° flexed elbow, a neutrally positioned forearm, a 0° to 30° flexed wrist, and an 0° to 30° deviated ulnar [10]. The handgrip strength was measured 3 times in each hand with at 1-minute intervals, and the average values were used for the analysis.

### 2.4. Laboratory Measurements

Blood samples were collected after an overnight fast of more than 8 h. TSH, fT4, total triiodothyronine (TT3), and TSH receptor antibody were measured at each visit. After the study was completed, free triiodothyronine (fT3) and total thyroxine (TT4) were measured with the stored blood at 0, 12, 26, and 52 weeks after the treatment. Serum TSH, fT4, and TT3 concentrations were measured by an Abbott Architect 2000 device (Abbott Diagnostics, Lake Forest, IL, USA) with chemiluminescent microparticle immunoassay method. Serum fT3 and TT4 concentrations were measured by radioimmunoassay (BRAHMS, Hennigsdorf, Germany) and electrochemiluminescence immunoassay (Roche, Mannheim, Germany), respectively. TSH receptor antibody was measured using an Elecsys® anti-TSH receptor antibody (Roche Diagnostics Ltd., Rotkreuz, Switzerland). Thyroxin-binding globulin (TBG) was measured using a radioimmunoassay kit (Brahms GmbH, Berlin, Germany). Total cholesterol, triglyceride, HDL cholesterol, aspartate aminotransferase (AST), alanine aminotransferase (ALT), and alkaline phosphatase (ALP) were measured, and LDL cholesterol was calculated.

To evaluate the thyroid homeostasis, peripheral deiodinase activity (${\hat{G}}_{D}$), thyroid's secretory capacity (${\hat{G}}_{T}$), and standardized TSH index (sTSHI) were calculated using the following formulas [11, 12]:

(1) $$\begin{array}{c} {\hat{G}}_{D}=\frac{8\times e^{-6}\times \left(5\times e^{-7}+\text{fT}4\right)\times \left(1+2\times e^{9}\times TBG\right)\times \text{fT}3}{0.026\times \text{fT}4}\;\left(reference\;range:20-60\;nmol/s\right),\\ {\hat{G}}_{T}=\frac{1.1\times 10^{-6}\times \left(2.75+TSH\right)\times TT4}{0.1\times TSH}\;\left(reference\;range:1.41-8.67\;pmol/s\right),\\ sTSHI=\frac{\ln\left(TSH+0.1345\times \text{fT}4\right)-2.7}{0.676}\;\left(reference\;range:-2\text{ to}+2\right). \end{array}$$

### 2.5. Statistical Analysis

Data are presented as the mean ± SD. To compare parameters before and after the treatment, the paired Wilcoxon test was performed. The correlation between thyroid hormones and REE was analyzed using Pearson's correlation coefficients. The change in food intake and muscle strength over time was analyzed using a linear mixed model. All statistical analyses were performed with SPSS version 22.0 for Windows software (SPSS, Chicago, IL, USA).

## 3. Results

### 3.1. Clinical Course and Laboratory Findings during the Antithyroid Drug Treatment

The mean age of the patients was 34 ± 14 years, and all were women. All patients preferred the antithyroid drug treatment and were treated with methimazole. The average starting dose was 23.3 ± 5.2 mg/day, and the doses were reduced according to the normalization of the thyroid function. Fifty-two weeks after the initial treatment, the dose was reduced to 6.7 ± 9.0 mg/day.

The changes in the biochemical parameters are shown in Table 1. Twenty-six weeks after the treatment, all patients had achieved a normal thyroid function: the thyroid hormone decreased gradually from week 4 to week 26, and TSH increased at a later time from week 12 to week 26 (Table 1). Their mean time to achieve euthyroidism was 17 ± 12 weeks. After a 52-week treatment of methimazole, the titer of anti-TSH receptor antibodies was decreased with marginal significance (17.99 ± 13.65 IU/L versus 4.43 ± 4.92 IU/L, *p* = 0.07).

Systolic blood pressure decreased significantly while diastolic blood pressure remained unchanged (Table 2). Heart rate also decreased significantly (Table 2). Although a beta-blocker was used in 2 patients until week 26 of the treatment, the heart rate remained decreased at week 52 of the treatment.

Liver enzymes including AST and ALT were slightly higher than normal at baseline but decreased to normal after the treatment (Table 1). Total cholesterol with both HDL and LDL cholesterols significantly increased after treatment, while triglyceride showed no change (Table 1). In addition, treatment for hyperthyroidism improved the quality of life (from 66 ± 12 scores at baseline to 76 ± 7 scores at week 52).

### 3.2. Changes in Body Weight and Dietary Intake

At the time of diagnosis, the mean body weight and BMI were 49.1 ± 3.3 kg and 20.5 ± 1.3 kg/m^2^, respectively. During the initial 8 weeks of treatment, the BMI significantly increased, reaching a peak at week 8, and then, it plateaued until week 26 (Table 2). At 52 weeks after the treatment, the BMI was slightly decreased but still higher than that at the baseline without any statistical significance. Although the hip circumference was slightly increased, the waist-to-hip ratio was not different after the treatment (Table 2).

**Figure 1:**
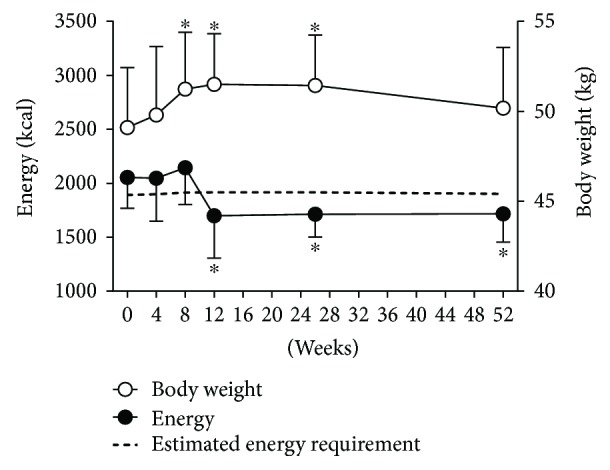
Change of total energy intake and body weight after antithyroid drug treatment. ^∗^*p* < 0.05 compared to baseline values.

Because a higher food intake with an increased appetite can be the major cause of weight gain, we first assessed the dietary energy intake using the three-day dietary record. At baseline, the mean total energy intake (2054 kcal) was higher than the estimated energy requirement (1891 kcal). However, the total energy intake rapidly decreased from week 8 to week 12, and then, it was maintained up to week 52 (*p* = 0.01; Figure 1). Consequentially, the mean total energy intake at 52 weeks (1716 kcal) was lower than the estimated energy intake (1903 kcal). There was no significant difference in the composition of macronutrients such as carbohydrates, proteins, and fats.

### 3.3. Resting Energy Expenditures (REE) and Thyroid Hormone

**Figure 2:**
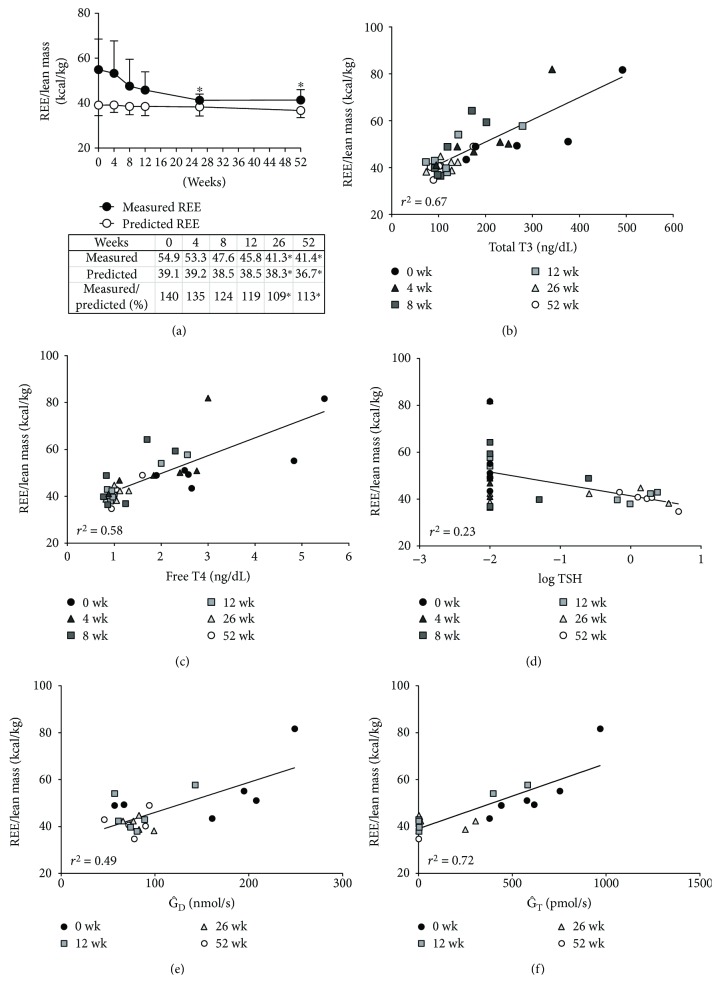
Change in REE according to the thyroid hormone levels and antithyroid drug treatment. (a) Change of REE after antithyroid drug treatment. (b) Correlation between REE and total T3. (c) Correlation between REE and free T4. (d) Correlation between REE and log TSH. (e) Correlation between REE and peripheral deiodinase activity (Ĝ_D_). (f) Correlation between REE and thyroid' secretory capacity (Ĝ_T_). ^∗^*p* < 0.05 compared to baseline values. REE, resting energy expenditure.

The discordance of the weight gain and decreased food intake led us to hypothesize that weight changes are mainly due to the changes in energy metabolism. To verify this hypothesis, lean mass-corrected REE was investigated during the antithyroid treatment. At the time of diagnosis, the measured REE in patients with Graves' disease was 40% higher than the predicted REE (54.9 ± 13.6 kcal/kg versus 39.1 ± 4.7 kcal/kg, *p* = 0.02; Figure 2(a)) in an untreated status. During the antithyroid treatment, the REE gradually decreased until week 52. The measured REE was 140% of the predicted REE at baseline and decreased to 113% at week 52 (*p* < 0.05; Figure 2(a)). The REE was positively correlated with the fT3 (*r*^2^ = 0.76, *p* < 0.01), TT3 (*r*^2^ = 0.67, *p* < 0.01; Figure 2(b)), and fT4 (*r*^2^ = 0.58, *p* < 0.01; Figure 2(c)) and reversely correlated with the log TSH (*r*^2^ = 0.23, *p* < 0.01; Figure 2(d)). Taken together, the increased REE according to the thyrotoxicosis is near completely rescued after the 52-week treatment following euthyroidism.

Furthermore, we investigated the association between REE and several parameters of thyroid homeostasis. Because deiodinase, a converting enzyme T4 to T3, mediates the metabolic function of thyroid hormone [13], we evaluated peripheral deiodinase activity (${\hat{G}}_{D}$) and thyroid's secretory capacity (${\hat{G}}_{T}$). The calculated peripheral deiodinase activity in untreated patients with hyperthyroidism was markedly increased by more than 2.5-fold compared to the known reference range (20−60 nmol/s) [12]. Fifty-two-week treatment of antithyroid drug significantly reduced peripheral deiodinase activity and thyroid's secretory capacity by 51% (*p* = 0.04) and 88% (*p* = 0.03), respectively (Table 1). More interestingly, peripheral deiodinase activity (*r*^2^ = 0.49, *p* < 0.01; Figure 2(e)) and thyroid's secretory capacity (*r*^2^ = 0.72, *p* < 0.01; Figure 2(f)) were positively correlated with REE. However, pituitary thyrotrophic function assessed by sTSHI did not change during treatment (from −2.3 ± 3.7 at baseline to −1.7 ± 2.6 at week 52, *p* = 0.80) and was not associated with REE (*r*^2^ = 0.03, *p* = 0.16).

### 3.4. Changes in Body Compositions

**Figure 3:**
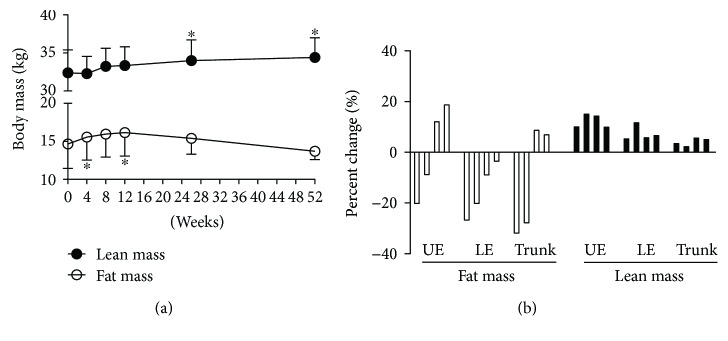
Change of body composition after antithyroid drug treatment. (a) Change of lean and fat mass measured by bioelectrical impedance after treatment. (b) Change of regional fat and lean mass measured by DXA after 52 weeks of treatment. UE, upper extremity; LE, lower extremity. ^∗^*p* < 0.05 compared to baseline values.

To further explore the compositional changes of the whole body, bioelectrical impedance analysis was performed. The body is largely composed of three components: fat mass, lean mass, and bone mineral content. As shown in Table 2, both lean mass and fat-free mass gradually increased until 52 weeks after the treatment: 4.9% (at 26 weeks, *p* < 0.05) and 6.2% (at 52 weeks, *p* < 0.05) increase in lean mass and 4.6% (at 26 weeks, *p* < 0.05) and 5.9% (at 52 weeks, *p* < 0.05) increase in fat-free mass. Interestingly, the maximum fat mass was observed at an earlier time week 12, while that of the lean mass was observed at a later time week 52 (Figure 3(a)). Both DXA at baseline and week 52 were available for four patients, and the change in regional fat and lean mass was observed (Figure 3(b)). The increment of lean mass in the arm (13 ± 3%) was the largest. Body fat (%) was also maximized at week 12 and then decreased to the baseline levels, but it was not statistically significant (Table 2).

**Figure 4:**
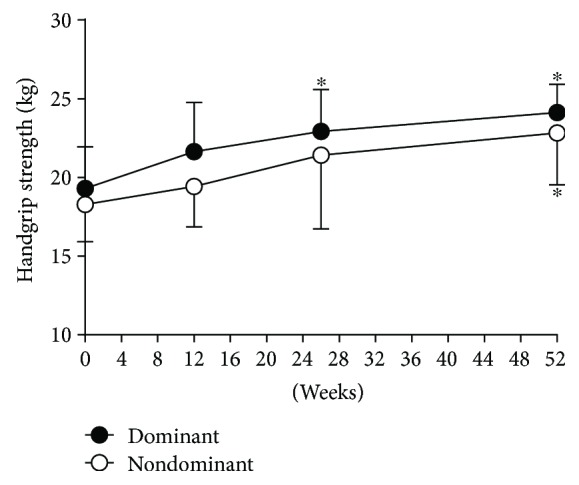
Change of handgrip strength in both dominant and nondominant hands after antithyroid drug treatment. ^∗^*p* < 0.05 compared to baseline values.

To assess whether the increased lean mass was accompanied by functional gain in muscle strength, the handgrip test was performed. Consistent with the lean mass changes, handgrip strength was significantly increased from 26 to 52 weeks after the treatment in both the dominant and nondominant hands (*p* < 0.01; Figure 4). There was no significant difference between the dominant and nondominant hands.

## 4. Discussion

The present study showed 3 key findings. First, treatment-related acute weight gain during the initial 6-month treatment of hyperthyroidism is reversed during the next 6 months with the recovery of the normal thyroid function. Second, the antithyroid treatment results in an increased body weight along with a decreased REE despite the decreased food intake. Third, the compositional analyses showed that fat mass increased during the initial 3 months and then reversed itself, while lean mass and muscle strength increased in the later 6 months suggesting body composition changes in a better direction. In the earlier period of the treatment for Graves' disease, the treatment-related increased body weight causes great concern in patients and might be a critical hurdle for medication adherence. The key findings in this study could reassure those concerns of Graves' patients.

The thyroid hormone is an important determinant of REE [1]. The treatment for hypothyroidism with levothyroxine was reported to increase the REE [14, 15]. Small changes in the dose of levothyroxine or target TSH levels could affect the REE in patients with hypothyroidism [15–17]. In healthy men, the administration of levothyroxine for 8 weeks increased the proteins related with the REE [18]. In consistent with previous reports [2, 3], the present study showed that the antithyroid treatment reduced the increased REE by hyperthyroidism following the increased body weight and compositional changes. Interestingly, although previous studies have shown strong correlations between the REE and fT3 [7, 19, 20], the present study firstly demonstrated that not only fT3 but also TT3 and fT4 were significantly correlated with the REE (Figure 2). Moreover, the present study demonstrated the positive correlations between thyroid hormone and REE in a wider range of thyroid function including hyperthyroidism (Figure 2) compared to the previous studies showing this correlation within the normal range [19, 20]. Several mechanisms have been proposed on how the thyroid hormone modulates the energy expenditure. The thyroid hormone can increase mitochondrial uncoupling resulting in an increase of thermogenesis and REE [21, 22]. In addition, the thyroid hormone, especially locally produced T3 by type 2 deiodinase, is essential for adaptive thermogenesis in brown adipose tissue [13]. It is well established that serum T3 concentration in patients with Graves' disease was disproportionately increased compared with serum T4 concentration [23]. Patients with Graves' disease have higher TT3/TT4 ratio or fT3/fT4 ratio suggesting an increased deiodinase activity [24, 25]. In this study, calculated deiodinase activity (${\hat{G}}_{D}$) was higher than the normal reference range [12]. Because Graves' disease is related to elevated TBG concentrations [26], an increased TBG as well as an increased deiodinase activity may have contributed to high ${\hat{G}}_{D}$ values. Interestingly, the peripheral deiodinase activity was significantly correlated with REE in our study.

One of the interesting findings in this study was that treatment of Graves' disease gradually increased muscle mass and improved muscle strength, while it also caused a transient gain in fat mass at an earlier time in the course of the treatment. Previous studies usually assessed the muscle mass with the DXA or bioelectrical impedance analysis and reported an increased lean mass [3, 4, 6]. The cross-sectional muscle area of the thigh determined by computed tomography showed an increased muscle mass, and Newton meters or dynamometer studies demonstrated enhanced muscle function after treatment of hyperthyroidism [27, 28]. The strength of this study is that the lean and fat mass and muscle strength were evaluated more intensively at multiple time points compared with previous studies. Based on our observations, we could reassure the patients that weight gain after treatment for hyperthyroidism mainly resulted from increased lean body mass.

The limitations of our study were the small sample size with the selected patients. Because this study was conducted only in six premenopausal Korean women, it is difficult to generalize our results to all genders and races. Besides, all the enrolled patients were well motivated for caring their health, which resulted in a limited weight gain compared to real-field patients. Therefore, the beneficial changes in body composition observed in this study might be restricted to those well-behaving patients. However, it is also reasonable to interpret that education on controlling dietary intake and exercise could be helpful for these patients. Further study is needed to investigate the effects of education on these patients.

## 5. Conclusions

Treatment for Graves' disease increased body weight and decreased the REE. However, the long-term changes in body composition moved in a beneficial direction increasing the lean body mass and muscle strength with the euthyroid status. These results might be used as reassuring evidence for patients who are concerned about the treatment-related weight gain in Graves' disease.

## Figures and Tables

**Table 1:** Change of biochemical parameter during 1 year of antithyroid treatment.

	Baseline	4 wk	8 wk	12 wk	26 wk	52 wk
Free T4 (ng/dL)	3.32 ± 1.46	**2.00 ± 0.87** ^∗^	**1.28 ± 0.61** ^∗^	**1.38 ± 0.72** ^∗^	**1.00 ± 0.22** ^∗^	**1.10 ± 0.25** ^∗^
Total T4 (*μ*g/dL)	16.0 ± 5.5			**7.6 ± 4.2** ^∗^	**6.2 ± 1.5** ^∗^	**7.8 ± 1.9** ^∗^
Free T3 (ng/dL)	2.21 ± 1.63			**0.57 ± 0.47** ^∗^	**0.38 ± 0.08** ^∗^	**0.40 ± 0.13** ^∗^
Total T3 (ng/dL)	295 ± 140	**206 ± 88** ^∗^	**131 ± 45** ^∗^	**137 ± 74** ^∗^	**109 ± 28** ^∗^	**107 ± 34** ^∗^
TSH (*μ*IU/mL)	0.01 ± 0.00	0.01 ± 0.00	0.06 ± 0.10	0.99 ± 0.99	3.30 ± 5.74	**1.74 ± 1.66** ^∗^
TSH receptor antibody (IU/L)	17.99 ± 13.65	14.13 ± 13.87	12.15 ± 12.79	10.97 ± 12.99	7.65 ± 9.43	4.43 ± 4.92
Peripheral deiodinase activity^a^ (${\hat{G}}_{D}$, nmol/s)	156 ± 78			**84 ± 31** ^∗^	79 ± 12	76 ± 17
Thyroid's secretory capacity^a^ (${\hat{G}}_{T}$, pmol/s)	623 ± 215			**165 ± 259** ^∗^	**95 ± 142** ^∗^	**76 ± 179** ^∗^
Total cholesterol (mg/dL)	158 ± 46			**210 ± 53** ^∗^	**203 ± 46** ^∗^	**221 ± 47** ^∗^
Triglyceride (mg/dL)	99 ± 54			85 ± 45	90 ± 34	96 ± 56
HDL cholesterol (mg/dL)	48 ± 9			**57 ± 9** ^∗^	**54 ± 11** ^∗^	**54 ± 11** ^∗^
LDL cholesterol (mg/dL)	90 ± 32			**136 ± 41** ^∗^	**131 ± 31** ^∗^	**148 ± 37** ^∗^
AST (IU/L)	48 ± 31	39 ± 20			**21 ± 8** ^∗^	**20 ± 7** ^∗^
ALT (IU/L)	78 ± 71	60 ± 51			**19 ± 15** ^∗^	**21 ± 24** ^∗^
ALP (IU/L)	101 ± 60	**118 ± 60** ^∗^			106 ± 51	62 ± 10

^∗^ *p* < 0.05 compared to baseline values; ^a^peripheral deiodinase activity (${\hat{G}}_{D}$) and thyroid' secretory capacity (${\hat{G}}_{T}$) were calculated with the equations described in Materials and Methods.

**Table 2:** Change of anthropometric parameter during 1 year of antithyroid treatment.

	Baseline	4 wk	8 wk	12 wk	26 wk	52 wk
Systolic BP (mmHg)	121 ± 11	**114 ± 11** ^∗^	115 ± 8	**106 ± 4** ^∗^	**108 ± 9** ^∗^	**107 ± 16** ^∗^
Diastolic BP (mmHg)	73 ± 9	72 ± 9	70 ± 10	66 ± 9	71 ± 9	67 ± 9
Heart rate	98 ± 21	95 ± 16	**84 ± 9** ^∗^	**83 ± 6** ^∗^	**81 ± 12** ^∗^	**81 ± 10** ^∗^
Weight (kg)	49.1 ± 3.3	49.8 ± 3.8	**51.2 ± 3.2** ^∗^	**51.5 ± 2.8** ^∗^	**51.4 ± 2.8** ^∗^	50.2 ± 3.4
BMI (kg/m^2^)	20.5 ± 1.3	20.8 ± 1.3	**21.5 ± 1.2** ^∗^	**21.5 ± 1.0** ^∗^	**21.5 ± 1.0** ^∗^	21.0 ± 0.7
Waist circumference (cm)	71 ± 4	70 ± 3	71 ± 6	73 ± 2	72 ± 4	70 ± 4
Hip circumference (cm)	90 ± 3	91 ± 4	**92 ± 2** ^∗^	**92 ± 2** ^∗^	91 ± 5	91 ± 2
Waist-to-hip ratio	0.79 ± 0.03	0.77 ± 0.02	0.77 ± 0.05	0.79 ± 0.01	0.80 ± 0.06	0.76 ± 0.04
Fat-free mass (kg)	34.5 ± 3.2	34.3 ± 2.4	35.3 ± 2.6	35.4 ± 2.6	**36.0 ± 2.9** ^∗^	**36.5 ± 2.7** ^∗^
Lean mass (kg)	32.4 ± 3.0	32.3 ± 2.3	33.2 ± 2.4	33.3 ± 2.5	**34.0 ± 2.7** ^∗^	**34.4 ± 2.6** ^∗^
Fat mass (kg)	14.7 ± 3.2	**15.6 ± 3.0** ^∗^	16.0 ± 3.0	**16.2 ± 3.1** ^∗^	15.4 ± 2.1	13.7 ± 1.1
Body fat (%)	29.8 ± 5.6	31.1 ± 4.3	31.0 ± 4.7	31.3 ± 4.9	29.9 ± 3.8	27.3 ± 1.5

^∗^ *p* < 0.05 compared to baseline values. BMI: body mass index; BP: blood pressure.
